# Supplementary material for: Transcriptomic timeseries links hepatic gene expression to an early and self-limited systemic response to enteric infection
Source: PLoS Pathog. 2026 Aug 5;22(8):e1014152. doi: 10.1371/journal.ppat.1014152 (PMC13466047; doi:10.1371/journal.ppat.1014152)
Supplement: Supplementary file 1 [file ppat.1014152.s001.docx]

**Fig. S1 *C. rodentium* immediately colonizes the cecum following inoculation.**

Extended data from Figure 1a. Colony-forming unit (CFU) in the cecum following inoculation with 5x10^9^ CFU. Circles represent data from individual animals, and the solid line represents the geometric mean. The open circle indicates that no bacteria were detected.
