# Supplementary material for: Transcriptomic timeseries links hepatic gene expression to an early and self-limited systemic response to enteric infection
Source: PLoS Pathog. 2026 Aug 5;22(8):e1014152. doi: 10.1371/journal.ppat.1014152 (PMC13466047; doi:10.1371/journal.ppat.1014152)
Supplement: Supplementary file 2 [file ppat.1014152.s002.docx]

**Fig. S2 Principal component scores of transcriptome.**

Extended data from Figure 1.

**AB.** Top 10 PC scores in the liver (A) or colon (B) from RNA-seq analysis are shown.

**CD.** Scree plot showing the fractional variance explained by each principal components (bar graph) for liver (C) or colonic epithelium (D) from RNA-seq analysis, and cumulative variance (line graph).
