# Supplementary material for: Transcriptomic timeseries links hepatic gene expression to an early and self-limited systemic response to enteric infection
Source: PLoS Pathog. 2026 Aug 5;22(8):e1014152. doi: 10.1371/journal.ppat.1014152 (PMC13466047; doi:10.1371/journal.ppat.1014152)
Supplement: Supplementary file 3 [file ppat.1014152.s003.docx]

**Fig. S3 Clusters of gene expression patterns in the liver and colonic epithelium during *C. rodentium* infection**

**A. B.** Heatmaps representing the z-score of genes differentially expressed over the course of the study in the liver (A) and colon (B). Differentially expressed genes compared to day 0, with the cut-off (log_2_ fold change > 1, adjusted p-value < 0.05) at least one time point, were included. The color code on the y-axis indicates groups identified by k-medoids clustering **The mouse ID on top matches the mouse ID in Fig. 1C and 1F. The panels on the right show the average z-score for genes within each cluster for individual animal at each time point. The symbols correspond to individual mouse IDs shown in the Fig. 1C and 1F.**
