# Supplementary material for: Transcriptomic timeseries links hepatic gene expression to an early and self-limited systemic response to enteric infection
Source: PLoS Pathog. 2026 Aug 5;22(8):e1014152. doi: 10.1371/journal.ppat.1014152 (PMC13466047; doi:10.1371/journal.ppat.1014152)
Supplement: Supplementary file 4 [file ppat.1014152.s004.docx]

**Fig. S4 Immunoprofiling in the liver, colonic lamina propria and epithelium**

**AB.** Flow cytometry plot of myeloid cells (A) and lymphocytes (B) analysis in the liver.

**CD.** Flow cytometry plot of myeloid cells (C) and lymphocytes (D) analysis in the colonic LP.

**EF.** Flow cytometry plot of myeloid cells (E) and lymphocytes (F) analysis in the colonic IEL.

**GH**. Flow cytometry plot of Memory CD4+ (G) and CD8+ (H) T cells in IEL fraction (top panels) and the line chart showing the fold changes compared to time-matched uninfected controls (bottom panels).
