# Supplementary material for: Transcriptomic timeseries links hepatic gene expression to an early and self-limited systemic response to enteric infection
Source: PLoS Pathog. 2026 Aug 5;22(8):e1014152. doi: 10.1371/journal.ppat.1014152 (PMC13466047; doi:10.1371/journal.ppat.1014152)
Supplement: Supplementary file 5 [file ppat.1014152.s005.docx]

**Fig. S5 PC1 gene expression in the liver in WT and Δ*eae* strain-infected animals**

Z-score of liver RNA expression of top 100 PC1 genes in uninfected control, wt-infected (*wt*), and Δ*eae*-infected *(*Δ*eae*) animals are shown in color. Uninfected control datasets and four wt-infected samples originate form time series transcriptome dataset. Z-score conversion was performed after data integration, batch correction, and normalization, as described in the Materials and methods.
