# Supplementary material for: Transcriptomic timeseries links hepatic gene expression to an early and self-limited systemic response to enteric infection
Source: PLoS Pathog. 2026 Aug 5;22(8):e1014152. doi: 10.1371/journal.ppat.1014152 (PMC13466047; doi:10.1371/journal.ppat.1014152)
Supplement: Supplementary file 6 [file ppat.1014152.s006.docx]

**Fig. S6 Liver cfu of Δ*espF* and wt *C. rodentium* strain 3 dpi**

Wt or Δ*espF C. rodentium* burden (CFU/g) in the liver on 3 days post inoculation. One-way ANOVA after lognormal conversion, and post-hoc test with Holm-Šídák's multiple comparisons.
