# Supplementary material for: Transcriptomic timeseries links hepatic gene expression to an early and self-limited systemic response to enteric infection
Source: PLoS Pathog. 2026 Aug 5;22(8):e1014152. doi: 10.1371/journal.ppat.1014152 (PMC13466047; doi:10.1371/journal.ppat.1014152)
Supplement: Supplementary file 7 [file ppat.1014152.s009.docx]

**Response to Editor and Reviewers**

Thank you for inviting us to submit a revised version of the manuscript.

The following is a summary of the new data and revisions to the figures.

1. Fig. 1B.	(DEGs number are added)
2. Fig. 3B-D. (Representative genes in each pathway are labeled)
3. New Fig. 5F. (CFU of wt and Δ*espF* strain in the liver)
4. New Fig. 5G. (qPCR analysis results of PC2 representative genes in the livers of animals infected with Δ*espF* mutant strains on 3 dpi)
5. New Fig. S5. (PC1 gene expression in the liver in WT and Δ*eae* strain-infected animals)
6. New Fig. S6. (Liver cfu of ΔespF and wt *C. rodentium* strain 3 dpi)

Below, we will respond point by point to the comments from the editor and reviewers:

**Additional Editor Comments :**

Two reviewer's have commented that the study is largely descriptive. Not every paper published needs to have a "mechanism", but the authors should address why their descriptive study is important to the field. Please pay attention to the other comments from the three reviewer's as well and address these experimentally, if possible.

Thank you for allowing us to submit the revised manuscript, and for acknowledging that “descriptive” work can advance our field. In the response to the reviewers, we outline how our study pushes the field forward and carefully respond to all the reviewers’ comments.

**Journal Requirements:**

1) Please ensure that the CRediT author contributions listed for every co-author are completed accurately and in full.

We checked that the CRediT contributions list is accurate and complete.

At this stage, the following Authors/Authors require contributions: Masataka Suzuki. Please ensure that the full contributions of each author are acknowledged in the "Add/Edit/Remove Authors" section of our submission form.
The list of CRediT author contributions may be found here: https://journals.plos.org/plospathogens/s/authorship#loc-author-contributions

Done.

2) We ask that a manuscript source file is provided at Revision. Please upload your manuscript file as a .doc, .docx, .rtf or .tex. If you are providing a .tex file, please upload it under the item type u2018LaTeX Source Fileu2019 and leave your .pdf version as the item type u2018Manuscriptu2019.

Done.

3) We do not publish any copyright or trademark symbols that usually accompany proprietary names, eg ©,  ®, or TM  (e.g. next to drug or reagent names). Therefore please remove all instances of trademark/copyright symbols throughout the text, including:
- ® on page: 28
- TM on page: 27.

Done.

4) We notice that your supplementary Figures are included in the manuscript file. Please remove them and upload them with the file type 'Supporting Information'. Please ensure that each Supporting Information file has a legend listed in the manuscript after the references list.

Done.

5) Thank you for stating "The processed RNA-seq data generated in this study have been deposited in the NCBI Gene Expression Omnibus (GEO) under accession number GSE326680. All raw sequences have been deposited in the NCBI Sequence Read Archive (SRA) under accession number PRJNA1445395." We strongly recommend all authors deposit their data before acceptance, as the process can be lengthy and hold up publication timelines. Please note that, though access restrictions are acceptable now, your entire minimal dataset will need to be made freely accessible if your manuscript is accepted for publication. This policy applies to all data except where public deposition would breach compliance with the protocol approved by your research ethics board.

All RNA-seq data has been deposited and will be available in GEO upon acceptance.

Note: If the reviewer comments include a recommendation to cite specific previously published works, please review and evaluate these publications to determine whether they are relevant and should be cited. There is no requirement to cite these works unless the editor has indicated otherwise.

**Reviewers' Comments:**

Reviewer's Responses to Questions

**Part I - Summary**
Please use this section to discuss strengths/weaknesses of study, novelty/significance, general execution and scholarship.

Reviewer #1: The authors compared temporal liver and gut responses using transcriptomics in the Citrobacter rodentium infection model. This approach provides a dynamic view of the gut–liver axis, in which low-level intestinal infection induces rapid hepatic responses, including acute-phase signalling and innate immune activation. By aligning transcriptional changes over time, the study offers insight into how local mucosal and systemic hepatic responses are temporally coordinated during infection.

Several previous studies have suggested that colonic immune responses remain largely localised and are not readily detected in the systemic circulation. In this context, the observation that early hepatic responses are systemically detectable represents an important contribution to the field.

The manuscript is well written, and the experimental work is of high quality. Although largely descriptive, the study addresses an important question and provides novel insights and a good resource.

Thank you for the thoughtful and positive critique of our findings. We agree that the paired kinetic analyses of transcriptional changes in the liver and colon along serum cytokine studies provides new insight into the control of systemic responses to enteric infection.

Reviewer #2: Thank you for the opportunity to review the manuscript by Hasegawa et al., titled “Transcriptomic timeseries links hepatic gene expression to an early and self-limited systemic response to enteric infection.” In this study, the authors used the enteric murine A/E pathogen Citrobacter rodentium to study inflammatory responses in the liver of infected mice, and how they are regulated, and temporally compare to responses in the colon. In brief, they identify an early, and self-limited burst of inflammatory signaling in the liver. In addition, they show that this response requires C. rodentium to be virulent (i.e., infect the intestinal epithelium).

Overall, this is a well written manuscript addressing a currently under-studied topic, i.e., the role of the liver in regulating the host response to an enteric bacterial infection. The authors focus on gene transcription (RNA seq), showing that the liver response to infection precedes that of the colonic epithelium, but rapidly reverses to baseline, as compared to progressive inflammatory/immune responses in the colon. Flow cytometry confirmed that this stronger colon response correlated with substantial immune cell infiltration into the infected colon. Serum proteins were also analyzed, while the authors showed that a C. rodentium mutant lacking intimin – and thus avirulent, did not trigger the same liver response. While the results the authors provide are potentially interesting, without some type of mechanistic experiments, the importance of these findings are uncertain.

We thank for the reviewer’s careful summary of our findings.

Reviewer #3: The present study by Hasegawa et al. focuses on gaining a better understanding of the timing of immune responses during enteric infection and the role that the liver can play in limiting uncontrolled pathogen expansion or damage. The results are interesting and reveal the role of the liver in mediating different immune responses at early, peak, and late time points. The manuscript can be improved by addressing the following comments.

Thank you for the positive assessment.

**Part II – Major Issues: Key Experiments Required for Acceptance**
Please use this section to detail the key new experiments or modifications of existing experiments that should be absolutely required to validate study conclusions.

Generally, there should be no more than 3 such required experiments or major modifications for a "Major Revision" recommendation. If more than 3 experiments are necessary to validate the study conclusions, then you are encouraged to recommend "Reject".

Reviewer #1: 1a. It would be helpful if the authors provided quantitative detail on the number of genes found to be differentially regulated within key pathways (e.g., KEGG categories). For example, the authors state that there was a greater number of differentially expressed genes (DEGs) in the liver compared to the colonic epithelium at early time points, how many genes are involved? In addition, which specific genes (not only pathways) were differentially expressed in the liver at 3 dpi.

We appreciate the comment.

a. To clarify the total number of DEGs on each day post infection, we added the specific counts of DEGs above the bars in Fig. 1B.

b. The identity of the specific genes differentially expressed in the liver 3 dpi is shown in Supplemental Table 2. In this table, the description and the number of genes in each pathway are presented in columns C and D. The number and specific genes found in that pathway are shown in columns L and M, respectively. The identities of DEGs, their log2 fold changes, and adjusted P-values are listed in Supplemental Table 1. Notably, as shown in Fig. 5 and 6, liver DEGs on 3 dpi include chemokines such as Ccl2, Ccl4, Ccl7, Cxcl1, and Cxcl2, and cytokines including Tnf.

We performed additional analysis using Enrich_KEGG, which is based on over-representation analysis, and created Table_Enrich_KEGG_DEG_number.xlsx listing the number of differentially expressed genes in KEGG pathways with p-values < 0.05 at each time point. We have added this data only in Zenodo (https://zenodo.org/records/21264234), because the number of genes in core enrichment in GSEA also represent the genes contributing to the enrichment therefore this table is redundant. The statement has been added in “Data availability” section.

1b. There appear to be overlaps in immune signalling pathways regulated in both the liver and colonic epithelium. The analysis would be strengthened if the authors clarified whether the same genes within these pathways are shared across tissues and time points, or whether distinct gene sets underlie these seemingly similar pathway enrichments.

We thank the reviewer for raising this excellent point. Figures 3B-D address this issue. Each row in these heatmaps shows the expression of an individual gene across time in both liver and colon. Each panel of heatmap shows the complete list of genes in the pathway. For example, Figure 3B shows the entire set of genes in the INTERFERON_ALPHA_RESPONSE pathway. The liver transcriptome data reveals that almost all genes in this pathway are activated 3-10 dpi. In contrast, in the colon, there is less comprehensive activation of the genes in this pathway. Thus, genes toward the bottom of the colonic heatmap have reduced expression on day 10, whereas they have increased expression in the liver at that time. Similarly, in panel 3C, the majority of genes in IL6_JAK_STAT3_ SIGNALING pathway are up-regulated between 3-10 dpi in the liver, while there is less comprehensive activation of genes in this pathway, and are partially down-regulated in the colon. These observations suggest that distinct regulatory processes mediate inflammatory responses to infection in these two tissues; we added this idea to the revised manuscript discussion lines 393-397.

We added the names of several key genes in each of the pathways in Fig. 3BCD, and additional information to the figure legend to provide clarification as follows: “The color of the “FoldChange” column represents the mean value of log2 fold change in the same sample group, and the color of “Zscore” column shows the value of the gene expression z-score of individual tissue samples. Each row of the heatmap depicts expression of the same gene across time points and tissues. Select gene names are labeled.”

2. It is somewhat surprising that metabolic genes were predominantly differentially expressed in the liver. This contrasts with prior reports demonstrating metabolic reprogramming in the colon during infection. The authors should discuss this in greater detail.

We agree with the reviewer that it is surprising that our gene expression studies did not reveal the same extent of metabolic reprogramming in the colon as observed in previous work. Instead, we discovered a more marked suppression of the expression of genes linked to metabolism in the liver than in the colon. Previous reports using quantitative proteomics by Berger (PMID:28988824) and Mullineaux-Sanders (PMID:35100877) showed that the abundance of proteins involved in metabolic pathways in the colonic epithelium was reduced at the peak of *C. rodentium* infection. In contrast, our bulk RNA-seq analyses of matched liver and colonic epithelium data suggested that metabolic signatures were reduced in the liver, particularly day 6 and 10 dpi, but the pattern in the colon was more variable. For example. Fig. 2BC shows that in the liver, expression of most genes in the OXIDATIVE_PHOSPHORYLATION and FATTY_ACID_METABOLISM pathways was reduced 6-10 dpi; at these times, in the colon, only about half of these genes showed reduced expression.

The discrepancies between the transcriptomic and proteomic analyses are unexpected and suggest post-transcriptional regulation of metabolic protein expression in the colon. We have added a discussion of this discrepancy in the revised discussion in line 416-419.

3. Hepatic CFU were not detected at 1–3 dpi or after 10 dpi, which is consistent with transient epithelial barrier disruption and subsequent repair at these time points. The eae mutant did not trigger hepatic responses (did it induce any colonic responses?). This is not unexpected, as the mutant does not attach to the mucosa and is therefore unlikely to be effectively sensed. While this serves as an important control, a more informative comparison might involve mutants such as espF or espF/map, which colonise at levels similar to wild type but do not disrupt tight junctions, a process that may be required for dissemination of luminal contents to the liver. Although repeating the full analysis with these mutants may not be practical, a targeted approach, similar to that shown for the eae mutant in Fig. 5B, would strengthen the conclusions.

The reviewer raises intriguing points that we address in new experiments. We created an isogenic *espF* deletion mutant strain by allelic exchange, and then infected wild-type animals with the mutant strain, and performed qPCR analysis of representative PC2 genes 3 dpi. At this time, 1 of 9 animals infected with wild-type *C. rodentium* and 1 of 9 Δ*espF*-infected animals had detectable CFU in the liver (new Fig. S6). The Δ*espF* strain had a mild defect in colonization in the colon 3 dpi (new Fig. 5F). Despite the mild colonization defect, the *espF* mutant lead to similar induction of PC2 genes as the wild type strain (new Fig. 5G). These results suggest that the disruption of the tight junction in the colon is not required to alter hepatic PC2 gene expression. These new results are now presented in the main text on lines (286-294) and in the discussion lines 355-359.

**New Fig. 5FG. Comparison of liver PC2 gene expression in mice infected with wild type vs Δ*espF* strain 3 dpi.**

**F.** Wt or Δ*espF* *C. rodentium* burden (CFU/g) in the colon on 3 days post inoculation. One-way ANOVA after lognormal conversion with Holm-Šídák's multiple comparisons test. Dotted line shows the limit of detection.

**G.** qPCR analysis of representative PC2 gene expression in the liver in animals infected with wild type (wt) or Δ*espF* *C. rodentium* 3 days post inoculation. Y-axis shows the relative value to *Gapdh* expression. Kruskal-Wallis test with Dunn’s multiple comparisons test.

Control (n = 6), wt-infected (n = 9), and Δ*espF*-infected (n = 9).

**New Fig. S6. Liver cfu of Δ*espF* and wt *C. rodentium* strain 3 dpi**

Wt or Δ*espF C. rodentium* burden (CFU/g) in the liver on 3 days post inoculation. One-way ANOVA after lognormal conversion, and post-hoc test with Holm-Šídák's multiple comparisons. Control (n = 6), wt-infected (n = 9), and Δ*espF*-infected (n = 9).

Reviewer #2: My one major concern is that the authors suggest they performed this study to uncover mechanisms that account for systemic responses to enteric infection. In fact, the manuscript is almost entirely descriptive. There are almost no mechanistic insights provided, aside from testing an avirulent strain of C. rodentium.

For the authors to argue that the transient liver response is important, they should explore what happens if this response is altered. I’m happy to be flexible on the exact approach they take, but options could include testing an innate immune deficient mouse (tlr4 deficient perhaps), or otherwise modifying the liver response to infection. Without such an experiment, the manuscript is almost entirely descriptive.

The reviewer raises a fair point. Our study is primarily descriptive. We have noted this weakness of the study in the text (e.g., we describe the correlative nature of the work in the abstract). However, we strongly believe that paired observations of the colon and liver responses, along with serum cytokines, from days 1 through 36 offers valuable new insights into systemic responses to enteric infection, as noted by Reviewers 1 and 3. Furthermore, the time-series design of our study enables a new understanding of the likely causal events driving systemic responses. This time-series design identified an earlier response in the liver than in the colon and provides correlational evidence that the liver is the primary driver of systemic responses to *C. rodentium* infection. We concede that the temporal data are not definitive, and we do not claim to have proved this hypothesis. We have added a limitation section to the discussion to further emphasize this issue.

To further address the reviewer’s concern, we did several additional experiments. As outlined above, in response to reviewer 1 point 3, we found that a Δ*espF* strain induced liver PC2 genes, revealing that the disruption of tight junction is not necessary for hepatic gene expression changes. In addition, we purchased Tlr2-/- and Tlr4-/- mice from JAX and performed RNA-seq analysis of liver tissue at 3 days post wild type *C. rodentium* infection (Figure R1 below). We observed that the expression of PC1 and PC2 genes in Tlr2-/- or Tlr4-/- liver was not as pronounced as observed in WT C57BL/6J animals, suggesting that these pattern recognition receptors are important for triggering hepatic gene expression 3 dpi.

**Figure R1. Tlr2 and Tlr4 modify hepatic responses to enteric infection.**

WT, Tlr2-/-, and Tlr4-/- mice were infected with wild-type *C. rodentium* (n = 4/genotype, batch 2), and their liver gene expression on 3 dpi was analyzed using RNA-seq. Data from uninfected WT animals and infected WT animals from time-series analysis (n = 4, left, batch 1) were combined, batch corrected, and normalized before z-score calculation. Heatmaps representing z-scores of gene expression of the top 100 PC1 (left) and PC2 (right) genes are shown. Each column represents individual samples and each row depicts expression of the same gene across genotype/condition.

We include these data exclusively in the response to reviewers because the animals used in this experiment were purchased from JAX and not littermates, and therefore not well controlled for microbiota effects. Our standard practice is to use products from heterozygous matings when studying the effects of host gene deficiencies. To create the mice properly controlling microbiota effects, would require an additional 6 months.

Reviewer #3: Would the authors clarify the rationale for using different sequencing platforms, fragment sizes, and the mixing of single and pair-end reads? It is not entirely clear whether all data were combined across experiments or whether the sequencing platforms and read types were balanced between groups. It'll also be helpful to discuss how Combat-seq was used and how effective it is for accounting for all these differences.

Although the datasets were generated on a single-organ basis using different sequencing platforms for time series analyses (Fig.1-3), multiple studies have demonstrated that differences in RNA-seq platforms or fragment sizes have little impact on the power to detect differentially expressed genes when the read length exceeds 50 bp. [PMID: 26100517, 28986563, 36741404, 31341269]. We have added supplemental information to Zenodo (https://zenodo.org/records/21264234) to clarify the sequencing platforms and conditions used for each sample.

Combat-seq was used only for Fig. 5D and new Fig. S5. Since we did not include uninfected condition in batch 2 (wt strain-infected vs. Δ*eae*-strain infected), we combined batch 1 dataset (time series analysis uninfected 0 dpi, and wt-infected 3 dpi) to confirm the activation of PC2 genes in wt-infected animals. Combat-seq was used to normalize for batch effects and the difference between pre- and post-Combat-seq batch correction is shown in the PCA plot below. The wt-infected animal group cluster closer after batch correction, suggesting that the Combat-seq effectively normalized for batch effects. Consequently, Fig. 5D and new Fig. S5 show similar pattern of PC1 and PC2 gene expression in wt-infected animals between two batches. We note, however, to avoid false-positive results caused by batch effects, we only carried out statistical analyses on data generate with the same platform.

**The effect of batch correction for Fig. 5D**

PCA plots shows the difference between pre- (left) and post-batch collection (right) using Combat-seq. Uninfected control 1-4 and wt-infected 1-4 samples originated from time series analysis 3 dpi (batch 1). wt-infected 5-8 and Δ*eae*-infected 1-4 samples originated from the second batch of RNA-seq experiment (batch 2). After applying Combat-seq, wt-infected samples clustered together, while Δ*eae*-infected samples kept distinct cluster to wt-infected and control samples.

Authors should provide more details about the RNAseq experiments, including RIN scores, sequencing depth, and general QC metrics such as mapping rates, rRNA contamination rates, and any pre-filtering used before DESeq2. For pathway enrichment analysis, I would encourage authors to report the specific database, version and/or collection used for the analysis.

We added the following sentences to the Method section “ RNA preparation from colonic epithelial cells and liver”, “RNA-seq and data analysis” and “Code availability”. We did not individually assess the mapping rate to rDNA; however, the high percentage of uniquely mapped reads strongly suggests that rRNA contamination was very low.

“The quality of all RNA samples was assessed using an Agilent TapeStation. The RNA integrity number (RIN) were above 8.0 for the liver samples, except for Liver-day10-1, which had a RIN of 7.3, and above 9.0 for the colon epithelium samples.”

“The average sequencing depth was approximately 40 million reads per sample, with a minimum of 20 million reads. More than 85% of reads were uniquely mapped, as assessed based on the STAR log reports.”

“Database and version information for the pathway analysis have been added to the top row of the Supplementary Table S2.”

“Code used for data analysis has been deposited and is available through Zenodo (https://zenodo.org/records/21264234).”

Previous research has established that escape into systemic organs is a major bottleneck during infection (Woodward et al. 2022). It is interesting that all mice used for RNA-seq were systemically colonized. Were the mice for RNA-seq analysis selected only if they showed liver colonization? If relevant, it would be helpful to also include the liver colonization rate across experiments.

We apologize for the confusion. We did not intentionally include only mice colonized with *C. rodentium* in the liver. For example, animal #1 at 10 dpi did not show liver colonization (Fig. 1A) but was a part of RNA-seq study. Also, we did not detect *C. rodentium* CFU in the liver of animals sampled on 1, 3, 17, 21, 36 dpi. The matched id information between CFU and RNA-seq data are provided in Fig. 1A, C and F and described in their respective figure legends. In addition, the ids shown on top of the heatmap in Fig. S3 also match the ids of Fig. 1A, C, and F.

**Part III – Minor Issues: Editorial and Data Presentation Modifications**
Please use this section for editorial suggestions as well as relatively minor modifications of existing data that would enhance clarity.

Reviewer #1: • Line 149 refers to Fig. 1I, which is missing from Fig. 1.

Thank you for pointing this out. We replaced the word “Fig. 1l” with “Fig. S3A”.

• On days 1 and 3 post-inoculation, C. rodentium was detected in the colon but not in the liver (Fig. 1A). At these time points, there were more DEGs in the liver than in the colonic epithelium (Fig. 1B, Supplementary Table 1). By contrast, from day 6 onwards, the number of colonic DEGs exceeded those in the liver, with the highest number observed at 10 dpi, corresponding to peak colonisation. Notably, the colonic transcriptional response remained elevated through 17 dpi, when bacterial burden was already declining, and thousands of DEGs persisted at 21–36 dpi, when most animals had cleared the infection. This prolonged response warrants further discussion.

Thank you for the comment. Mullineaux-Sanders et al, have conducted time series proteomics of colonic epithelial cells, and found that interferon gamma-mediated MHCII expression persist even after 48 dpi (PMID: 35100877). Similarly, we found that activation of interferon gamma signaling persist after 36 dpi.We expanded the discussion about persistent colonic transcriptomic change in line 387-388.

Reviewer #2: The authors use standard approaches to isolate epithelial cells from the mouse colon, but there will likely be at least modest contamination by non-epithelial cells. This should be acknowledged.

We acknowledge that there is potential contamination of intraepithelial lymphocytes and stromal cells localized underneath the epithelium. We included the sentence that “FACS analysis suggested that the majority of the cells in this epithelial fraction were Epcam-positive (epithelial cells), but some intraepithelial immune cells were also detected. In principle, there could be contamination from immune cells and stromal cells derived from the mucosal lamina propria.’ in the method line 485-488.

Reviewer #3: The authors used N = 5 mice for profiling C. rodentium burden but only N = 4 for RNA-seq. Could you briefly clarify why the sample sizes differ between the analyses?

Since we have two tissue samples with eight time points from 5 animals (in total of 80 samples), we randomly included 4 samples from each condition for the RNA-seq analysis to save the sequencing cost without compromising the statistical robustness.

Can authors further clarify in the legend and methods whether the DEGs were calculated relative to their corresponding t0 or uninfected controls?

We included the sentence “compared to uninfected day 0” in the legend for Fig. 1B, and “ Differentially expressed genes were identified using the R-package DESeq2 (v1.36.0) with an adjusted *P* < 0.05 and an absolute log2 fold change of 1 compared to uninfected time 0 control animals,” in “RNA-seq and data analysis” in the method section.

Line 451 states that an absolute fold change of 1 was used. Could the authors clarify whether this is correct or whether they used an absolute log2 fold change of 1?

Thank you for pointing out this error. We used absolute log2 fold change of 1. We corrected the sentence.

Were the N = 8 mice used for qPCR different than the ones used for RNAseq? Additionally, given the heterogeneous responses observed across experiments, it would be interesting to discuss whether this variability is correlated with intestinal bacterial burden or liver colonization levels (if data is available)

We appreciate the reviewer’s suggestion. Yes, these animals were different than the ones used for RNA-seq. To address this point, we performed the correlation analysis between CFU vs. PC2 gene expression in the new experiment requested by reviewer 1. In WT *C. rodentium* infected animals (n = 9), there is no clear correlation between colon or liver CFU vs. cytokine expression (Figure below, correlation matrix), while Cxcl2, Ccl2, Ccl4, and Ccl7 expression significantly correlated each other. These result suggest that *C. rodentium* burden in the colon and liver does not explain liver PC2 gene expression.

**Correlation matrix**

Log10-converted CFU and relative gene expression value were used for Spearman’s correlation analysis. The color shows correlation coefficient r and the values on the heatmap shows *P*-values. Only *P*-values <0.05 are shown.

Could the authors further clarify why they chose to only focus on the top 100 PC2 genes for the ∆eae strain in Figure 5D? It would be informative to determine whether PC1 genes or any other relevant DEGs related to Figures 2 and 3 are relevant for the ∆eae strain.

This is excellent point. We found PC2 genes most intriguing because their expression was self-limiting and correlated with systemic cytokines. We created a new Fig. S5 to show PC1 genes in Δ*eae*-infected animals. Similar to PC2, the *eae* mutant did not stimulate PC1 genes.

**New Fig. S5 PC1 gene expression in the liver in WT and Δ*eae* strain-infected animals**

Z-score of liver RNA expression of top 100 PC1 genes in uninfected control, wt-infected (*wt*), and Δ*eae*-infected *(*Δ*eae*) animals are shown in color. Uninfected control datasets and four wt-infected samples originate form time series transcriptome dataset. Z-score conversion was performed after data integration, batch correction, and normalization.

.

Authors should provide more methodological details for qPCR experiments, including the endogenous controls used, the number of replicates, the statistical methods used, and primer efficiencies, if relevant.

We added the details in the Figure 5B legend and qPCR method section.

The legends or methods for Figures 4 and 6 do not include any information about the number of samples/mice used for the analysis (apologies if it is, and I missed it).

Thank you for pointing out the missing information. FACS analysis for Figure 4 used 5 animals per group for each time point and it is described in the last sentence of the legend: n = 5/group. Figure 6 represents the mean values of z-score from 4 animals per each time point (n = 4/Days). We added the information in the legend.

I believe the manuscript would benefit by expanding the introduction to further introduce the reader to some of the concepts that appear later in the paper.

We added to the introduction as suggested, however, we prefer a style that introduces new concepts where they are relevant to the results.

Can the authors briefly discuss what would be the role of the cecum in the proposed mechanism? It is known that the cecum is the initial site of colonization of C. rodentium.
The reviewer raises an interesting point. It is possible that early cecal colonization is an important trigger of early liver gene expression. We now include this point in “Limitations” section.

To improve reproducibility, I would encourage the authors to make their analysis code publicly available on GitHub or Zenodo.

Thank you for the suggestion. We posted our bulk RNA-seq analysis code in Zenodo (<https://zenodo.org/records/21264234>) and stated in “Code availability” section. The code will become publicly available upon acceptance.
