# Supplementary material for: Transcriptomic timeseries links hepatic gene expression to an early and self-limited systemic response to enteric infection
Source: PLoS Pathog. 2026 Aug 5;22(8):e1014152. doi: 10.1371/journal.ppat.1014152 (PMC13466047; doi:10.1371/journal.ppat.1014152)
Supplement: Peer review history

# Peer review: Decision Letter 0

**DOI:** 10.1371/journal.ppat.1014152.r001

**Authors:** Leigh Knodler, Thomas Guillard

14 May 2026

Transcriptomic timeseries links hepatic gene expression to an early and self-limited systemic response to enteric infection

PLOS Pathogens

Dear Dr. Suzuki,

Thank you for submitting your manuscript to PLOS Pathogens. After careful consideration, we feel that it has merit but does not fully meet PLOS Pathogens's publication criteria as it currently stands. Therefore, we invite you to submit a revised version of the manuscript that addresses the points raised during the review process.

Please submit your revised manuscript by Jul 13 2026 11:59PM. If you will need more time than this to complete your revisions, please reply to this message or contact the journal office at plospathogens@plos.org. When you're ready to submit your revision, log on to https://www.editorialmanager.com/ppathogens/ and select the 'Submissions Needing Revision' folder to locate your manuscript file.

Please include the following items when submitting your revised manuscript:

* A letter that responds to each point raised by the editor and reviewer(s). You should upload this letter as a separate file labeled 'Response to Reviewers'. This file does not need to include responses to any formatting updates and technical items listed in the 'Journal Requirements' section below.

* A marked-up copy of your manuscript that highlights changes made to the original version. You should upload this as a separate file labeled 'Revised Manuscript with Track Changes'.

* An unmarked version of your revised paper without tracked changes. You should upload this as a separate file labeled 'Manuscript'.

We look forward to receiving your revised manuscript.

Kind regards,

Leigh Knodler

Academic Editor

PLOS Pathogens

Thomas Guillard

Section Editor

Editor-in-Chief

PLOS Pathogens

orcid.org/0000-0003-2946-9497

Michael Malim

Editor-in-Chief

PLOS Pathogens

orcid.org/0000-0002-7699-2064

**Additional Editor Comments :**

Two reviewer's have commented that the study is largely descriptive. Not every paper published needs to have a "mechanism", but the authors should address why their descriptive study is important to the field. Please pay attention to the other comments from the three reviewer's as well and address these experimentally, if possible.

**Journal Requirements:**

1) Please ensure that the CRediT author contributions listed for every co-author are completed accurately and in full.

At this stage, the following Authors/Authors require contributions: Masataka Suzuki. Please ensure that the full contributions of each author are acknowledged in the "Add/Edit/Remove Authors" section of our submission form.

The list of CRediT author contributions may be found here: https://journals.plos.org/plospathogens/s/authorship#loc-author-contributions

2) We ask that a manuscript source file is provided at Revision. Please upload your manuscript file as a .doc, .docx, .rtf or .tex. If you are providing a .tex file, please upload it under the item type u2018LaTeX Source Fileu2019 and leave your .pdf version as the item type u2018Manuscriptu2019.

3) We do not publish any copyright or trademark symbols that usually accompany proprietary names, eg ©,  ®, or TM  (e.g. next to drug or reagent names). Therefore please remove all instances of trademark/copyright symbols throughout the text, including:

- ® on page: 28

- TM on page: 27.

4) We notice that your supplementary Figures are included in the manuscript file. Please remove them and upload them with the file type 'Supporting Information'. Please ensure that each Supporting Information file has a legend listed in the manuscript after the references list.

5) Thank you for stating "The processed RNA-seq data generated in this study have been deposited in the NCBI Gene Expression Omnibus (GEO) under accession number GSE326680. All raw sequences have been deposited in the NCBI Sequence Read Archive (SRA) under accession number PRJNA1445395." We strongly recommend all authors deposit their data before acceptance, as the process can be lengthy and hold up publication timelines. Please note that, though access restrictions are acceptable now, your entire minimal dataset will need to be made freely accessible if your manuscript is accepted for publication. This policy applies to all data except where public deposition would breach compliance with the protocol approved by your research ethics board.

Note: If the reviewer comments include a recommendation to cite specific previously published works, please review and evaluate these publications to determine whether they are relevant and should be cited. There is no requirement to cite these works unless the editor has indicated otherwise.

**Reviewers' Comments:**

Reviewer's Responses to Questions

**Part I - Summary**

Please use this section to discuss strengths/weaknesses of study, novelty/significance, general execution and scholarship.

Reviewer #1: The authors compared temporal liver and gut responses using transcriptomics in the Citrobacter rodentium infection model. This approach provides a dynamic view of the gut–liver axis, in which low-level intestinal infection induces rapid hepatic responses, including acute-phase signalling and innate immune activation. By aligning transcriptional changes over time, the study offers insight into how local mucosal and systemic hepatic responses are temporally coordinated during infection.

Several previous studies have suggested that colonic immune responses remain largely localised and are not readily detected in the systemic circulation. In this context, the observation that early hepatic responses are systemically detectable represents an important contribution to the field.

The manuscript is well written, and the experimental work is of high quality. Although largely descriptive, the study addresses an important question and provides novel insights and a good resource.

Reviewer #2: Thank you for the opportunity to review the manuscript by Hasegawa et al., titled “Transcriptomic timeseries links hepatic gene expression to an early and self-limited systemic response to enteric infection.” In this study, the authors used the enteric murine A/E pathogen Citrobacter rodentium to study inflammatory responses in the liver of infected mice, and how they are regulated, and temporally compare to responses in the colon. In brief, they identify an early, and self-limited burst of inflammatory signaling in the liver. In addition, they show that this response requires C. rodentium to be virulent (i.e., infect the intestinal epithelium).

Overall, this is a well written manuscript addressing a currently under-studied topic, i.e., the role of the liver in regulating the host response to an enteric bacterial infection. The authors focus on gene transcription (RNA seq), showing that the liver response to infection precedes that of the colonic epithelium, but rapidly reverses to baseline, as compared to progressive inflammatory/immune responses in the colon. Flow cytometry confirmed that this stronger colon response correlated with substantial immune cell infiltration into the infected colon. Serum proteins were also analyzed, while the authors showed that a C. rodentium mutant lacking intimin – and thus avirulent, did not trigger the same liver response. While the results the authors provide are potentially interesting, without some type of mechanistic experiments, the importance of these findings are uncertain.

Reviewer #3: The present study by Hasegawa et al. focuses on gaining a better understanding of the timing of immune responses during enteric infection and the role that the liver can play in limiting uncontrolled pathogen expansion or damage. The results are interesting and reveal the role of the liver in mediating different immune responses at early, peak, and late time points. The manuscript can be improved by addressing the following comments.

**********

**Part II – Major Issues: Key Experiments Required for Acceptance**

Please use this section to detail the key new experiments or modifications of existing experiments that should be absolutely required to validate study conclusions.

Generally, there should be no more than 3 such required experiments or major modifications for a "Major Revision" recommendation. If more than 3 experiments are necessary to validate the study conclusions, then you are encouraged to recommend "Reject".

Reviewer #1: 1a. It would be helpful if the authors provided quantitative detail on the number of genes found to be differentially regulated within key pathways (e.g., KEGG categories). For example, the authors state that there was a greater number of differentially expressed genes (DEGs) in the liver compared to the colonic epithelium at early time points, how many genes are involved? In addition, which specific genes (not only pathways) were differentially expressed in the liver at 3 dpi.

1b. There appear to be overlaps in immune signalling pathways regulated in both the liver and colonic epithelium. The analysis would be strengthened if the authors clarified whether the same genes within these pathways are shared across tissues and time points, or whether distinct gene sets underlie these seemingly similar pathway enrichments.

2. It is somewhat surprising that metabolic genes were predominantly differentially expressed in the liver. This contrasts with prior reports demonstrating metabolic reprogramming in the colon during infection. The authors should discuss this in greater detail.

3. Hepatic CFU were not detected at 1–3 dpi or after 10 dpi, which is consistent with transient epithelial barrier disruption and subsequent repair at these time points. The eae mutant did not trigger hepatic responses (did it induce any colonic responses?). This is not unexpected, as the mutant does not attach to the mucosa and is therefore unlikely to be effectively sensed. While this serves as an important control, a more informative comparison might involve mutants such as espF or espF/map, which colonise at levels similar to wild type but do not disrupt tight junctions, a process that may be required for dissemination of luminal contents to the liver. Although repeating the full analysis with these mutants may not be practical, a targeted approach, similar to that shown for the eae mutant in Fig. 5B, would strengthen the conclusions.

Reviewer #2: My one major concern is that the authors suggest they performed this study to uncover mechanisms that account for systemic responses to enteric infection. In fact, the manuscript is almost entirely descriptive. There are almost no mechanistic insights provided, aside from testing an avirulent strain of C. rodentium.

For the authors to argue that the transient liver response is important, they should explore what happens if this response is altered. I’m happy to be flexible on the exact approach they take, but options could include testing an innate immune deficient mouse (tlr4 deficient perhaps), or otherwise modifying the liver response to infection. Without such an experiment, the manuscript is almost entirely descriptive.

Reviewer #3: Would the authors clarify the rationale for using different sequencing platforms, fragment sizes, and the mixing of single and pair-end reads? It is not entirely clear whether all data were combined across experiments or whether the sequencing platforms and read types were balanced between groups. It'll also be helpful to discuss how Combat-seq was used and how effective it is for accounting for all these differences.

Authors should provide more details about the RNAseq experiments, including RIN scores, sequencing depth, and general QC metrics such as mapping rates, rRNA contamination rates, and any pre-filtering used before DESeq2. For pathway enrichment analysis, I would encourage authors to report the specific database, version and/or collection used for the analysis.

Previous research has established that escape into systemic organs is a major bottleneck during infection (Woodward et al. 2022). It is interesting that all mice used for RNA-seq were systemically colonized. Were the mice for RNA-seq analysis selected only if they showed liver colonization? If relevant, it would be helpful to also include the liver colonization rate across experiments.

**********

**Part III – Minor Issues: Editorial and Data Presentation Modifications**

Please use this section for editorial suggestions as well as relatively minor modifications of existing data that would enhance clarity.

Reviewer #1: • Line 149 refers to Fig. 1I, which is missing from Fig. 1.

• On days 1 and 3 post-inoculation, C. rodentium was detected in the colon but not in the liver (Fig. 1A). At these time points, there were more DEGs in the liver than in the colonic epithelium (Fig. 1B, Supplementary Table 1). By contrast, from day 6 onwards, the number of colonic DEGs exceeded those in the liver, with the highest number observed at 10 dpi, corresponding to peak colonisation. Notably, the colonic transcriptional response remained elevated through 17 dpi, when bacterial burden was already declining, and thousands of DEGs persisted at 21–36 dpi, when most animals had cleared the infection. This prolonged response warrants further discussion.

Reviewer #2: The authors use standard approaches to isolate epithelial cells from the mouse colon, but there will likely be at least modest contamination by non-epithelial cells. This should be acknowledged.

Reviewer #3: The authors used N = 5 mice for profiling C. rodentium burden but only N = 4 for RNA-seq. Could you briefly clarify why the sample sizes differ between the analyses?

Can authors further clarify in the legend and methods whether the DEGs were calculated relative to their corresponding t0 or uninfected controls?

Line 451 states that an absolute fold change of 1 was used. Could the authors clarify whether this is correct or whether they used an absolute log2 fold change of 1?

Were the N = 8 mice used for qPCR different than the ones used for RNAseq? Additionally, given the heterogeneous responses observed across experiments, it would be interesting to discuss whether this variability is correlated with intestinal bacterial burden or liver colonization levels (if data is available)

Could the authors further clarify why they chose to only focus on the top 100 PC2 genes for the ∆eae strain in Figure 5D? It would be informative to determine whether PC1 genes or any other relevant DEGs related to Figures 2 and 3 are relevant for the ∆eae strain.

Authors should provide more methodological details for qPCR experiments, including the endogenous controls used, the number of replicates, the statistical methods used, and primer efficiencies, if relevant.

The legends or methods for Figures 4 and 6 do not include any information about the number of samples/mice used for the analysis (apologies if it is, and I missed it).

I believe the manuscript would benefit by expanding the introduction to further introduce the reader to some of the concepts that appear later in the paper.

Can the authors briefly discuss what would be the role of the cecum in the proposed mechanism? It is known that the cecum is the initial site of colonization of C. rodentium.

To improve reproducibility, I would encourage the authors to make their analysis code publicly available on GitHub or Zenodo.

**********

PLOS authors have the option to publish the peer review history of their article (what does this mean?). If published, this will include your full peer review and any attached files.

If you choose “no”, your identity will remain anonymous but your review may still be made public.

**Do you want your identity to be public for this peer review?**  For information about this choice, including consent withdrawal, please see our Privacy Policy.

Reviewer #1: No

Reviewer #2: No

Reviewer #3: No

[NOTE: If reviewer comments were submitted as an attachment file, they will be attached to this email and accessible via the submission site. Please log into your account, locate the manuscript record, and check for the action link "View Attachments". If this link does not appear, there are no attachment files.]

**Figure resubmission:**

**Reproducibility:**

?>

# Author comment: Author response to Decision Letter 1

**DOI:** 10.1371/journal.ppat.1014152.r002

15 Jul 2026

Attachment Submitted filename: Response_to_Reviewers.docx

# Peer review: Decision Letter 1

**DOI:** 10.1371/journal.ppat.1014152.r003

**Authors:** Leigh Knodler, Thomas Guillard

26 Jul 2026

Dear Dr. Waldor,

We are pleased to inform you that your manuscript 'Transcriptomic timeseries links hepatic gene expression to an early and self-limited systemic response to enteric infection' has been provisionally accepted for publication in PLOS Pathogens.

Before your manuscript can be formally accepted you will need to complete some formatting changes, which you will receive in a follow up email. A member of our team will be in touch with a set of requests.

Please note that your manuscript will not be scheduled for publication until you have made the required changes, so a swift response is appreciated.

IMPORTANT: The editorial review process is now complete. PLOS will only permit corrections to spelling, formatting or significant scientific errors from this point onwards. Requests for major changes, or any which affect the scientific understanding of your work, will cause delays to the publication date of your manuscript.

Should you, your institution's press office or the journal office choose to press release your paper, you will automatically be opted out of early publication. We ask that you notify us now if you or your institution is planning to press release the article. All press must be co-ordinated with PLOS.

Thank you again for supporting Open Access publishing; we are looking forward to publishing your work in PLOS Pathogens.

Best regards,

Leigh Knodler

Academic Editor

PLOS Pathogens

Thomas Guillard

Section Editor

PLOS Pathogens

Sumita Bhaduri-McIntosh

Editor-in-Chief

PLOS Pathogens

orcid.org/0000-0003-2946-9497

Michael Malim

Editor-in-Chief

PLOS Pathogens

orcid.org/0000-0002-7699-2064

***********************************************************

Reviewer Comments (if any, and for reference):

Reviewer's Responses to Questions

**Part I - Summary**

Please use this section to discuss strengths/weaknesses of study, novelty/significance, general execution and scholarship.

Reviewer #1: I have no further comments and congratulate the authors on a job well done

Reviewer #2: N/A

**********

**Part II – Major Issues: Key Experiments Required for Acceptance**

Please use this section to detail the key new experiments or modifications of existing experiments that should be absolutely required to validate study conclusions.

Generally, there should be no more than 3 such required experiments or major modifications for a "Major Revision" recommendation. If more than 3 experiments are necessary to validate the study conclusions, then you are encouraged to recommend "Reject".

Reviewer #1: (No Response)

Reviewer #2: N/A

**********

**Part III – Minor Issues: Editorial and Data Presentation Modifications**

Please use this section for editorial suggestions as well as relatively minor modifications of existing data that would enhance clarity.

Reviewer #1: (No Response)

Reviewer #2: N/A

**********

PLOS authors have the option to publish the peer review history of their article (what does this mean?). If published, this will include your full peer review and any attached files.

If you choose “no”, your identity will remain anonymous but your review may still be made public.

**Do you want your identity to be public for this peer review?**  For information about this choice, including consent withdrawal, please see our Privacy Policy.

Reviewer #1: **Yes:**  Gad Frankel

Reviewer #2: No

# Editor report: Acceptance letter

**DOI:** 10.1371/journal.ppat.1014152.r004

**Authors:** Leigh Knodler, Thomas Guillard

Dear Dr. Waldor,

We are delighted to inform you that your manuscript, "Transcriptomic timeseries links hepatic gene expression to an early and self-limited systemic response to enteric infection," has been formally accepted for publication in PLOS Pathogens.

We have now passed your article onto the PLOS Production Department who will complete the rest of the pre-publication process. All authors will receive a confirmation email upon publication.

The corresponding author will soon be receiving a typeset proof for review, to ensure errors have not been introduced during production. Please review the PDF proof of your manuscript carefully, as this is the last chance to correct any scientific or type-setting errors. Please note that major changes, or those which affect the scientific understanding of the work, will likely cause delays to the publication date of your manuscript. Note: Proofs for Front Matter articles (Pearls, Reviews, Opinions, etc...) are generated on a different schedule and may not be made available as quickly.

Soon after your final files are uploaded, the early version of your manuscript, if you opted to have an early version of your article, will be published online. The date of the early version will be your article's publication date. The final article will be published to the same URL, and all versions of the paper will be accessible to readers.

For Research Articles, you will receive an invoice from PLOS for your publication fee after your manuscript has reached the completed accept phase. If you receive an email requesting payment before acceptance or for any other service, this may be a phishing scheme. Learn how to identify phishing emails and protect your accounts at https://explore.plos.org/phishing.

Thank you again for supporting open-access publishing; we are looking forward to publishing your work in PLOS Pathogens.

Best regards,

Sumita Bhaduri-McIntosh

Editor-in-Chief

PLOS Pathogens

orcid.org/0000-0003-2946-9497

Michael Malim

Editor-in-Chief

PLOS Pathogens

orcid.org/0000-0002-7699-2064
